# Clinical evaluation of two commercial PCR kits for the detection of nonviral sexually transmitted infections

**DOI:** 10.1099/jmm.0.002037

**Published:** 2025-07-03

**Authors:** Alexandre Gaudin, Nadège Hénin, Marie Gardette, Cécile Bébéar, Sabine Pereyre

**Affiliations:** 1 Bacteriology Department, National Reference Centre for Bacterial Sexually Transmitted Infections, Bordeaux University Hospital, Bordeaux, France; 2 CNRS UMR 5234 Fundamental Microbiology and Pathogenicity, University of Bordeaux, Bordeaux, France

**Keywords:** *Chlamydia trachomatis*, *Mycoplasma genitalium*, *Neisseria gonorrhoeae*, *Trichomonas vaginalis*, bacterial sexually transmitted infections, real-time multiplex PCR

## Abstract

**Introduction.** Sexually transmitted infections (STIs) are a worldwide health issue with a high number of asymptomatic cases and the possibility of multiple infections.

**Gap statement.** New multiplex real-time PCR kits targeting pathogens involved in nonviral STIs are regularly launched, but only some of them have been evaluated in comparative studies.

**Aim.** This study evaluated the clinical performance of two multiplex real-time PCR commercial kits for the detection of *Chlamydia trachomatis*, *Neisseria gonorrhoeae*, *Mycoplasma genitalium* and *Trichomonas vaginalis*: the Bosphore STD Urethritis Mini Bundle Kit (BK; Anatolia Geneworks) and the Viasure Sexually Transmitted Disease Real-Time PCR Detection Kit (VK; CerTest BIOTEC).

**Methodology.** A total of 240 clinical specimens were evaluated. The results were compared with those of the Cobas CT/NG and TV/MG kits (Roche Diagnostics), used as reference methods.

**Results.** Positive agreement ranged between 83.3% and 87.8% for the detection of *C. trachomatis*, *N. gonorrhoeae* and *T. vaginalis* using validated specimen types. For *M. genitalium* detection, positive agreement was 83.0% for the BK and 68.1% for the VK, which missed 31.9% of *M. genitalium*-positive specimens. Negative agreement ranged between 98.4% and 100% across the targeted micro-organisms. Both kits were easy to use and compatible with several DNA extraction and PCR thermal cyclers. The VK also detected the genital commensal bacteria *Ureaplasma* spp. and *Mycoplasma hominis*, which should not be targeted in STI detection kits.

**Conclusion.** Both kits are convenient methods and showed good performance for the detection of nonviral STIs, but users should be aware of a lower sensitivity of the VK for the detection of *M. genitalium*.

## Introduction

Sexually transmitted infections (STIs) are a worldwide issue. *Chlamydia trachomatis*, *Neisseria gonorrhoeae*,*Treponema pallidum* and *Trichomonas vaginalis* caused 374 million new symptomatic cases per year among people aged 15–49 years, according to the World Health Organization, with *T. vaginalis* being the most prevalent nonviral STI pathogen [1]. The prevalence of *Mycoplasma genitalium* infection is 1–2% in the general population of high-income countries [2,4] but increases to up to 16.7% in high-risk populations, such as pre-exposure prophylaxis (PrEP) users [5,6].

STIs represent an important challenge because of their high incidence, the high rate of asymptomatic patients, the possibility of concurrent infections and the adverse impacts on public health. Multiplex PCR assays may be a convenient method to quickly detect STI pathogens in a single step with high sensitivity and specificity. New multiplex real-time PCR kits targeting pathogens involved in nonviral STIs are regularly launched, but only some of them have been evaluated in comparative studies [7,11].

To assist clinical diagnostic laboratories in selecting kits that offer both optimal performance and suitability for routine use, we evaluated the clinical performance of two multiplex real-time PCR commercial kits for the detection of *C. trachomatis*, *N. gonorrhoeae*, *M. genitalium* and *T. vaginalis*: the Bosphore STD Urethritis Mini Bundle Kit (BK; Anatolia Geneworks) and the Viasure Sexually Transmitted Disease Real-Time PCR Detection Kit (VK; CerTest BIOTEC). Their performance was compared with that of the Cobas CT/NG and Cobas TV/MG kits (Roche Diagnostics, USA), used as reference methods [12,18].

## Methods

### Clinical specimens

Between August 2022 and February 2023, remnants of clinical swab specimens collected in Cobas PCR medium (Roche Molecular Systems, USA) and first-void urine received at the Bacteriology Department of Bordeaux University Hospital (Bordeaux, France) were prospectively collected and stored at −80 °C until testing with commercial kits. The aim of the sample selection process was to systematically enrol the first 40 samples detected as positive for *C. trachomatis*, *N. gonorrhoeae* or *M. genitalium* using the Cobas CT/NG and Cobas TV/MG kits; as many samples as possible positive for *T. vaginalis* using the Cobas TV/MG kit; and 70 samples negative for all 4 pathogens using both Cobas kits. A total of 22 *T*. *vaginalis*-positive samples, collected between January and July 2022, were also retrospectively included to increase the number of samples positive for this parasite.

### DNA extraction and amplification using commercial kits

**Fig. 1.**
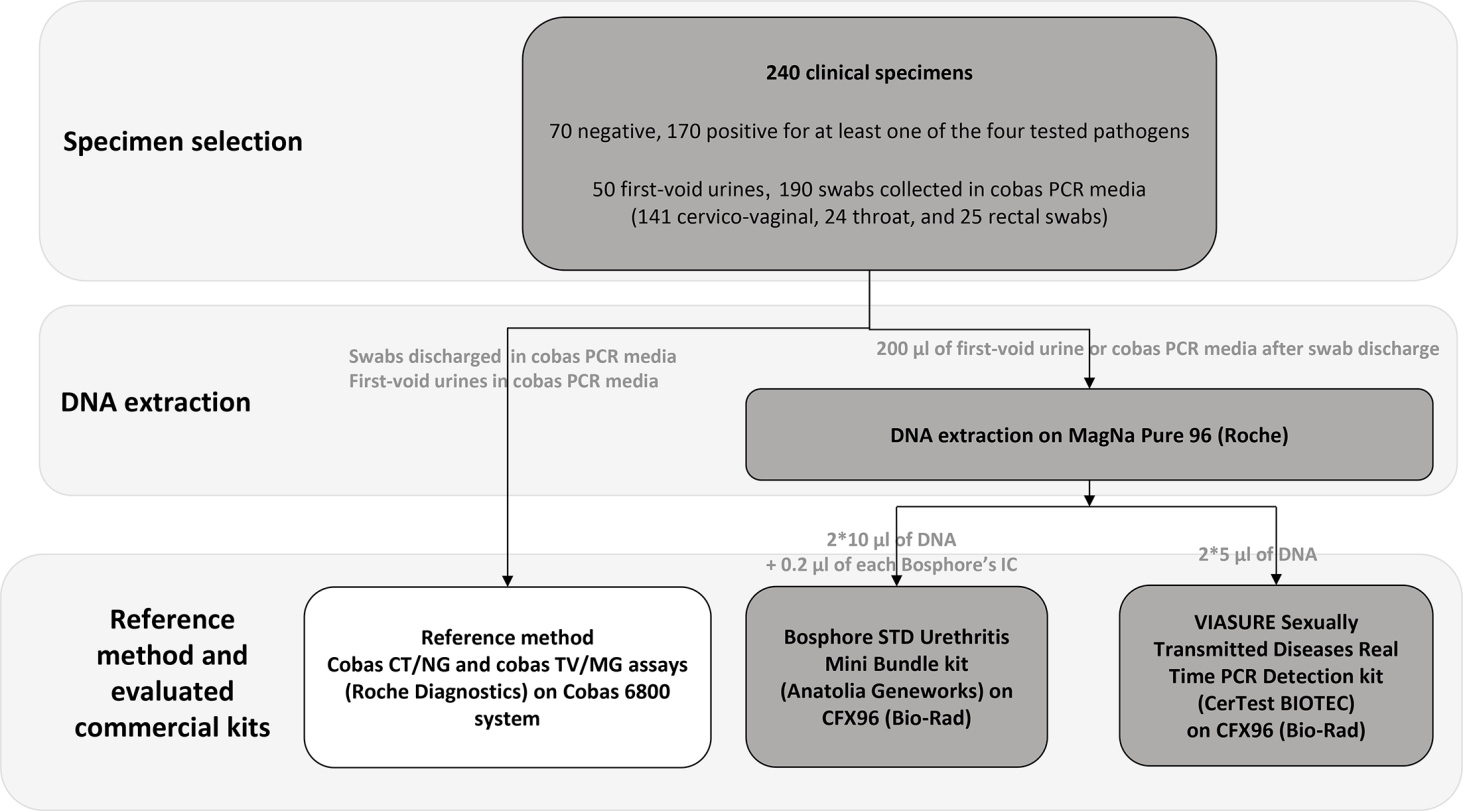
Flowchart of the study.

Nucleic acids were extracted from 200 µl clinical samples using the MagNA Pure 96 DNA and viral NA small-volume kit and the MagNA Pure 96 instrument (Roche Diagnostics) yielding a DNA extract of 100 µl (Fig. 1). The BK provides one internal control for each test panel, which can be added before or after the extraction step. Here, 0.2 µl of each internal control was added to the DNA extracts. Two 10 µl and two 5 µl aliquots of DNA were used to run the BK and VK, respectively (Fig. 1).

Both commercial kits are CE-IVD-marked and were run according to the manufacturer’s instructions. They both employ Taq-polymerase technology, and their main characteristics are presented in Table 1.

**Table 1.** Main characteristics of the two tested kits as well as the Cobas reference kits

Kit	Cobas® CT/NGCobas® TV/MG (Roche Diagnostics)	BK (Anatolia Geneworks)	VK (CerTEST Biotec)
**Pathogens (detected targets**)	CT (*OmpA* gene and cryptic plasmid)NG (NG DR-9 A and B sequences)TV (1 undisclosed multi-copy target)MG (2 undisclosed targets)	CT, NG, TV and MG (undisclosed targets)	CT (*Orf2* plasmidic region)NG (*porA* and *opa* genes)TV (*T*. *vaginalis*-specific 2 kb repeated sequence)MG (MgPa adhesin gene)MH (*yidC* gene)UU and UP (urease gene)
**Validated specimen types**	CT and NG: urine, cervical, rectal, throat and vaginal swabs and cervical samplesMG and TV: urine, cervical, urethral and vaginal swabs	Urine, tissue/biopsy, semenCervicovaginal, urethral and vaginal swabs	CT and MG: urine, urethral and vaginal swabsNG: urine, rectal, urethral and vaginal swabsTV: urethral and vaginal swabsMH and UU: urine, rectal, urethral and vaginal swabsUP: urine, urethral and vaginal swabs
**Limits of detection** (manufacturer’s published information)	CT: 40 elementary bodies/mlNG: 1 c.f.u. /mlTV: 0.16 cell /mlMG: 3.2 copies/ml	CT: 3 copies/reactionNG: 22 fg/reactionTV: 32 fg/reactionMG: 17 copies/reaction	≥10 DNA copies/reaction
**Input sample volume**	Urine: 1.2 mlPreservCyt and swabs: 1 ml	10 µl DNA for each panel	5 µl DNA for each panel
**Internal control**	Included	Included, one in each panel	Included
**Positive control**	Included for each pathogen	Included for each pathogen	Included for each pathogen
**Negative control**	Included	Included	Included
**Number of test/kit**	CT/NG: 480TV/MG: 384	25, 50 or 100 depending on the kit reference	12 eight-well strips per kit
**Total test time**	~1 h 30/run	~4 h /run*	~3 h /run*
**Number of reactions/runs**	96-well plate	96-well plate	96-well plate or 8-well strip
**Thermal cycler used in this study**	Cobas® 6800 System (Roche Diagnostics)	CFX96® Touch Real-Time PCR System (Bio-Rad)	CFX96® Touch Real-Time PCR System (Bio-Rad)
**Data analysis software**	Cobas® 6800/8800 Software System v1.4 (Roche)	CFX Maestro 1.1 v4.1.2 (Bio-Rad)	CFX Maestro 1.1 v4.1.2 (Bio-Rad)
**List price/reaction, excluding taxes†**	28.27€ each	13.12€	56.25€

*DNA extraction was performed using the MagNA Pure 96 (Roche) and took ~1 h and 30 min.

†DNA extraction price is not included.

Because the two commercial kits were tested on specimens after a few days of storage at −80 °C, both Cobas kits were rerun on specimens for which both evaluated commercial kits failed to detect the expected pathogens. The Cobas initial positive results were confirmed in all cases.

### Data analysis

The Cobas CT/NG and Cobas TV/MG kits were used as reference methods for *C. trachomatis* and *N. gonorrhoeae* detection and *M. genitalium* and *T. vaginalis* detection, respectively.

Overall percentage agreement (OPA), positive percentage agreement (PPA) and negative percentage agreement (NPA) were calculated along with the corresponding 95% confidence intervals (CIs) and Cohen’s kappa (*κ*) for the OPA.

## Results

### Characteristics of included samples

In total, 240 specimens were collected, with 59.6% from women and 40.4% from men (Fig. 1, Tables 2, S1 and S2, available in the online Supplementary Material). The specimens included 141 cervicovaginal swabs, 50 first-void urines, 25 rectal swabs and 24 throat swabs, reflecting the sample types commonly received in diagnostic laboratories for non-viral STI screening.

**Table 2.** Specimen types according to sex

Specimen type	Male	Female	Total (%)
First-void urine	49	1	50 (20.8)
Cervicovaginal swabs	–	141	141 (58.8)
Rectal swabs	24	1	25 (10.4)
Throat swabs	24	0	24 (10.0)
Total (%)	97 (40.4)	143 (59.6)	240 (100)

The mean age of the patients was 32 years (28 years for women and 38 years for men), and the median age was 28 years (26 years for women and 33 years for men). Among the 240 specimens included, 61 were positive for *C. trachomatis*, and 50 were positive for *N. gonorrhoeae*, using the Cobas CT/NG kit; and 52 were positive for *M. genitalium*, and 44 were positive for *T. vaginalis*, using the Cobas TV/MG reference kit (Table S1). In all, 31 specimens were positive for 2 distinct pathogens. Seventy samples were negative for the four pathogens using both Cobas kits.

The two evaluated PCR kits yielded no invalid samples.

### Performance results using validated specimen types

For *C. trachomatis* detection, the PPA and NPA were 85.4% and 100%, respectively, for both kits, with a *κ* coefficient of 0.90 (Table 3). Each kit missed seven samples detected as positive using the Cobas CT/NG reference kit. Among these seven samples, five (four cervicovaginal swabs and one first-void urine) were falsely detected as *C. trachomatis*-negative by both kits. Regarding *N. gonorrhoeae* detection, the PPA ranged between 83.3% and 87.5%, and the NPA ranged between 98.4% and 100%, with no statistically significant difference between the kits (Table 3). Late fluorescence was nevertheless detected by the VK for one cervicovaginal sample and two rectal samples [cycle threshold (Ct) of 41.3, 40.3 and 43, respectively], but the *N. gonorrhoeae* detection result was negative according to the manufacturer’s instructions. For *M. genitalium* detection, the PPA for the BK was 83.0%. It was only 68.1% for the VK, but the difference between the kits was not statistically significant as their CIs overlapped (Table 3). Nevertheless, the VK missed 31.9% (15/47) of the *M. genitalium*-positive samples detected using the Cobas MG/TV kit (13 cervicovaginal samples and 2 first-void urines). NPA was 99.3% for both kits. Regarding *T. vaginalis* detection, the PPA ranged between 86.4% and 87.8%, while the NPA was 100% for both kits, with a *κ* coefficient of 0.91.

**Table 3.** Clinical performance of the two commercial kits using validated sample types

Pathogen	Evaluated kit	No. of validated samples tested	Positive with reference		Negative with reference		Overall % agreement (95% CI)	Positive % agreement (95% CI)	Negative % agreement (95% CI)	*κ*
			Positive	Negative	Positive	Negative				
**CT**	BK	191	41	7	0	143	96.3 (92.6–98.2)	85.4 (72.8.–92.8)	100 (97.4–100)	0.90
	VK	191	41	7	0	143	96.3 (92.6–98.2)	85.4 (72.8–92.8)	100 (97.4–100)	0.90
**NG**	BK	191	15	3	0	173	98.4 (95.5–99.5)	83.3 (60.8–94.2)	100 (97.8–100)	0.90
	VK	216	28	4	3	181	96.8 (93.5–98.4)	87.5 (71.9–95.0)	98.4 (95.3–99.4)	0.87
**MG**	BK	191	39	8	1	143	95.3 (91.3–97.5)	83.0 (69.9–91.1)	99.3 (96.2–99.9)	0.87
	VK	191	32	15	0	144	92.1 (87.4–95.2)	68.1 (53.8–79.6)	100 (97.4–100)	0.76
**TV**	BK	191	38	6	0	147	96.9 (93.3–98.6)	86.4 (73.3–93.6)	100 (97.5–100)	0.91
	VK	141	36	5	0	100	96.5 (92.0–98.5)	87.8 (74.5–94.7)	100 (96.3–100)	0.91

### Performance according to sample type

Another analysis was separately performed on the 141 cervicovaginal swabs, the 50 first-void urines, the 25 rectal swabs and the 24 throat swabs (Tables S3–S6). In cervicovaginal swab samples (Table S3), performance was in accordance with the results obtained when using validated specimens, presented in Table 3.

Regarding first-void urine (Table S4), *T. vaginalis* was poorly detected by both kits. The VK, which has not been validated for *T. vaginalis* detection in urine, yielded a PPA of 33.3%, whereas the BK yielded a PPA of 0% as the kit detected none of the three *T. vaginalis*-positive urine samples.

Rectal swabs are not approved for use with either of the evaluated commercial kits, except the VK for the detection of *N. gonorrhoeae*. In rectal swabs, the PPAs for *C. trachomatis* detection were lower overall than those observed in validated sample types, with a PPA of only 72.7% for the VK (Tables 3 and S5).

Regarding throat swabs (Table S6), which have not yet undergone validation testing for either kit, the PPA for *N. gonorrhoeae* detection was only 50.0% and 55.6% for the BK and VK, respectively. A low NPA of 66.7% was also observed for the BK for the detection of *T. vaginalis*.

### Performance results when using samples positive for two distinct micro-organisms

Table 4 shows the PPA of the two kits depending on whether a single or two distinct STI micro-organisms were present in the specimens. Only validated sample types were considered in this analysis. Using the BK, the PPA decreased for *C. trachomatis*, *N. gonorrhoeae* and *T. vaginalis* detection when two pathogens were present compared with only one, from 92.9% to 72.2%, 100% to 66.7% and 90.9% to 66.7%, respectively, but the decreases were not significant as the CIs overlapped. Using the VK, the PPA decreased from 89.3% to 77.8% for *C. trachomatis* detection only.

**Table 4.** Performance of the two kits using validated sample types when samples contained one versus two micro-organisms

Evaluated kit	Pathogen	Positive % agreement (95% CI) in samples positive for one STI micro-organism	Positive % agreement (95% CI) in samples positive for two STI micro-organisms
**BK**	CT	92.9 (77.4–98.0)	72.2 (49.1–87.5)
	NG	100 (67.6–100)	66.7 (35.4–87.9)
	MG	80.6 (65.0–90.2)	90.0 (59.6–98.2)
	TV	90.9 (76.4–96.9)	66.7 (35.4–87.9)
**VK**	CT	89.3 (72.8–96.3)	77.8 (54.8–91.0)
	NG	80.0 (54.8–93.0)	93.3 (70.2–98.8)
	MG	66.7 (50.3–79.8)	70.0 (39.7–89.2)
	TV	87.5 (71.9–95.0)	85.7 (48.7–97.4)

## Discussion

Multiplex PCR can simultaneously detect several micro-organisms that are difficult to culture or not culturable from samples. It may also detect micro-organisms that were not initially suspected and facilitate rapid and appropriate treatment.

In this study, we evaluated the clinical performances of two multiplex real-time PCR commercial kits for the detection of STI pathogens. In comparative studies, the reference used plays a critical role in the interpretation of the results. In this study, as in our previous one [11], we used the Cobas CT/NG and Cobas TV/MG kits as reference assays; these kits have proven good performance for the detection of *C. trachomatis*, *N. gonorrhoeae*, *M. genitalium* and *T. vaginalis* on cohorts of thousands of samples in several publications [7,12,18].

The two evaluated kits had OPA >96% for the detection of *C. trachomatis*, *N. gonorrhoeae* and *T. vaginalis* and only 92.1–95.3% for *M. genitalium* (Table 3). Notably, the PPA was only 68.1% for *M. genitalium* detection using the VK, suggesting that this kit would miss a large number of *M. genitalium*-positive samples that would have been detected using a Cobas kit. This lack of sensitivity for *M. genitalium* detection has already been reported for other multiplex PCR kits targeting STI agents [11] and may be due to the 100-fold lower bacterial load in *M. genitalium*-positive specimens compared with *C. trachomatis*-positive ones [19]. This may also be related to multiplex PCR having less sensitivity for any given target.

Indeed, in this study, several PPA values were lower in specimens containing two STI micro-organisms compared to those with a single pathogen (Table 4). Although the difference was not statistically significant, as the CIs overlapped, the BK showed lower PPA for the detection of *C. trachomatis*, *N. gonorrhoeae* and *T. vaginalis* when two pathogens were present. This inconsistent performance of the BK in detecting multiple infections has important implications for real-world STI diagnosis, where patients are often co-infected.

Regarding the VK, a previous study evaluated its performance using the Allplex STI7 Essential Assay kit (Seegene, South Korea) as the reference assay [20]. The PPA ranged between 93.3% and 100% depending on the targeted micro-organism; our values were much lower, at 68.1–87.8%, using the Cobas kits as references. This may be due to the high sensitivity of the Cobas kits [7,12,18]. Nevertheless, using both reference assays, a low sensitivity was found for *M. genitalium* detection.

The BK and VK are convenient kits because they are compatible with many DNA extractors and thermocyclers. The possibility of using 8-well unit strips or a 96-well plate with the BK allows routine operations to be adapted to the number of samples. The turnaround time is similar for the two kits, but the PCR run duration is ~1 h and 30 min for the VK and 2 h and 30 min for the BK. Regarding the interpretation of the VK results, the manufacturer recommends that results be considered positive only if the amplification Ct is below 40. This was an issue for 16 samples (4 *C*. *trachomatis-*, 3 *N*. *gonorrhoeae-*, 7 *M*. *genitalium-* and 2 *T*. *vaginalis*-positive samples), which had Ct values over 40 and thus had to be interpreted as negative but were in fact positive for these pathogens. If these samples had been interpreted as positive, the PPAs for *C. trachomatis*, *N. gonorrhoeae*, *M. genitalium* and *T. vaginalis* detection would have increased to 93.8%, 96.9%, 83% and 88.6%, instead of 85.4%, 87.5%, 68.1% and 87.8%, respectively. However, dealing with high Ct values (Ct>40) in PCR assays also carries a risk of false-negative results. Nevertheless, feedback on Ct values could help commercial kit manufacturers refine their detection thresholds by aligning them with reference standards and continuously improve kit performance.

Regarding the routine use of both kits, a major drawback is that throat and rectal swabs have not been approved for use with these kits, except for rectal swabs for the detection of *N. gonorrhoeae* using the VK. This can be an issue when testing populations such as men who have sex with men and PrEP users because the detection of these STI pathogens often requires testing multiple sites simultaneously, including genital, pharyngeal and rectal sites [21].

In addition, the BK also detected *Mycoplasma hominis*, *Ureaplasma parvum* and *Ureaplasma urealyticum*, which are common, commensal species that colonize the urogenital tract of both sexes, although *U. urealyticum* may rarely cause non-gonococcal urethritis in men [22,24]. According to the International Union Against Sexually Transmitted Infection (IUSTI), routine testing is not recommended for these bacteria [22]. In the context of increasing antimicrobial resistance, particularly for *N. gonorrhoeae* and *M. genitalium* (23–25), the use of kits detecting these bacteria should be avoided.

This study has some limitations. First, while Cobas assays were used as reference assays, they are not gold standard methods despite their previously reported strong performance (7, 12–18). Therefore, only OPA, PPA and NPA were considered in this study instead of sensitivity and specificity. Additionally, Sanger sequencing was not used to resolve discrepant samples, as its sensitivity is often lower than that of real-time PCR, leading to a potential risk of falsely categorizing positive samples as negative, which may have impacted the performance evaluation of the tested commercial kits. Finally, metagenomics could have been used as the reference method; however, its cost for 240 samples was unfortunately beyond the scope of this comparative study of commercial kits. Second, the experiments were not conducted simultaneously, and a limited number of positive throat and rectal samples were used. Third, there is a low prevalence of *T. vaginalis* infection in France (3), so we had to retrospectively add *T. vaginalis*-positive specimens to our study. Finally, our sole focus was on comparing the performances of two commercial kits; we did not intend to determine the prevalences of the four pathogens.

In conclusion, the BK and VK are convenient methods for the detection of non-viral STI. Both kits showed good performance for the detection of *C. trachomatis*, *N. gonorrhoeae* and *T. vaginalis,* but the VK showed a lower sensitivity for *M. genitalium* detection.

## Supplementary material

10.1099/jmm.0.002037 Uncited Supplementary Material 1.

## Abbreviations

BK: Bosphore® STD Urethritis Mini Bundle Kit
CI: confidence interval
CT: *Chlamydia trachomatis*
Ct: cycle threshold
MG: *Mycoplasma genitalium*
MH: *Mycoplasma hominis*
NG: *Neisseria gonorrhoeae*
NPA: negative percentage agreement
OPA: overall percentage agreement
PPA: positive percentage agreement
PrEP: pre-exposure prophylaxis
STI: sexually transmitted infection
TV: *Trichomonas vaginalis*
UP: *Ureaplasma parvum*
UU: *Ureaplasma urealyticum*
VK: Viasure® Sexually Transmitted Disease Real-Time PCR Detection Kit
