# Supplementary material for: Clinical evaluation of two commercial PCR kits for the detection of nonviral sexually transmitted infections
Source: J Med Microbiol. 2025 Jul 3;74(7):002037. doi: 10.1099/jmm.0.002037 (PMC12231094; doi:10.1099/jmm.0.002037)
Supplement: Uncited Supplementary Material 1. [file jmm-74-02037-s001.pdf]

Table S1. Co-infections among the 240 tested samples

Sample status	Number (%) of samples
CT-, NG-, MG-, and TV-negative	70 (29.2)
MG-positive	38 (15.8)
CT-positive	34 (14.2)
TV-positive	33 (13.8)
NG-positive	31 (12.9)
CT-, and NG-positive	13 (5.4)
CT-, and TV-positive	6 (2.5)
CT-, and MG-positive	5 (2.1)
MG-, and NG-positive	4 (1.7)
MG-, and TV-positive	3 (1.3)
CT-, MG-, and NG-positive	1 (0.4)
CT-, MG-, and TV-positive	1 (0.4)
CT-, NG-, and TV-positive	1 (0.4)

CT, *C. trachomatis*; NG, *N. gonorrhoeae* ; MG, *M. genitalium* ; TV, *T. vaginalis*.

Table S2. Clinical performance of the two evaluated commercial kits for 240 tested samples compared to the reference kits (Cobas CT/NG and Cobas TV/MG)

Pathogens	Evaluated kits	No. of valid samples	Positive with reference		Negative with reference		Overall % agreement [95%CI]	Positive % agreement [95%CI]	Negative % agreement [95%CI]	κ
			Positive	Negative	Positive	Negative				
**CT**	BK	240	52	9	0	179	96.3 [93.0-98.0]	85.2 [74.3-92.0]	100 [97.9-100]	0.90
	VK	240	51	10	0	179	95.8 [92.5-97.7]	83.6 [72.4-90.8]	100 [97.9-100]	0.88
**NG**	BK	240	37	13	0	190	94.6 [91.0-96.8]	74.0 [60.4-84.1]	100 [98.0-100]	0.82
	VK	240	38	12	3	187	93.8 [89.9-96.2]	76.0 [62.6-85.7]	98.4 [95.5-99.5]	0.80
**MG**	BK	240	43	9	2	186	95.4 [92.0-97.4]	82.7 [70.3-90.6]	98.9 [96.2-99.7]	0.86
	VK	240	35	17	0	188	92.9 [89.0-95.5]	67.3 [53.8-78.5]	100 [98.0-100]	0.76
**TV**	BK	240	38	6	9	187	93.8 [89.9-96.2]	86.4 [73.3-93.6]	95.4 [91.5-97.6]	0.80
	VK	240	37	7	0	196	97.1 [94.1-98.6]	84.1 [70.6-92.1]	100 [98.1-100]	0.90

BK, Bosphore® STD Urethritis Mini Bundle kit; VK, Viasure® Sexually Transmitted Diseases Real Time PCR Detection kit.
CI, confidence interval, CT, *C. trachomatis*, NG, *N. gonorrhoeae*, MG, *M. genitalium*, TV, *T. vaginalis*.

Table S3. Clinical performance of the two evaluated commercial kits for 141 cervico-vaginal swabs compared to the reference kits (Cobas CT/NG and Cobas TV/MG)

Pathogens	Evaluated kits	No. of validated samples	Positive with reference		Negative with reference		Overall % agreement [95%CI]	Positive % agreement [95%CI]	Negative % agreement [95%CI]	κ
			Positive	Negative	Positive	Negative				
**CT**	BK	141	37	6	0	98	95.7 [91.0-98.0]	86.0 [72.7-93.4]	100 [96.2-100]	0.90
	VK	141	37	6	0	98	95.7 [91.0-98.0]	86.0 [72.7-93.4]	100 [96.2-100]	0.90
**NG**	BK	141	11	3	0	127	97.9 [93.9-99.3]	78.6 [52.4-92.4]	100 [97.1-100]	0.87
	VK	141	12	2	2	125	97.2 [92.9-98.9]	85.7 [60.1-96.0]	98.4 [94.4-99.6]	0.84
**MG**	BK	141	31	6	1	103	95.0 [90.1-97.6]	83.8 [68.9-92.3]	99.0 [94.8-99.8]	0.87
	VK	141	24	13	0	104	90.8 [84.9-94.5]	64.9 [48.8-78.2]	100 [96.4-100]	0.73
**TV**	BK	141	38	3	0	100	97.9 [93.9-99.3]	92.7 [80.6-97.5]	100 [96.3-100]	0.95
	VK	141	36	5	0	100	96.5 [92.0-98.5]	87.8 [74.5-94.7]	100 [96.3-100]	0.91

BK, Bosphore® STD Urethritis Mini Bundle kit; VK, Viasure® Sexually Transmitted Diseases Real Time PCR Detection kit.
CI, confidence interval; CT, *C. trachomatis*; NG, *N. gonorrhoeae*; MG, *M. genitalium*; TV, *T. vaginalis*.

Table S4. Clinical performance of the two evaluated commercial kits for 50 first-void urine samples compared to the reference kits (Cobas CT/NG and Cobas TV/MG)

**Pathogens**	**Evaluated kits**	**No. of validated samples**	**Positive with reference**		**Negative with reference**		**Overall % agreement [95%CI]**	**Positive % agreement [95%CI]**	**Negative % agreement [95%CI]**	**κ**
			**Positive**	**Negative**	**Positive**	**Negative**				
**CT**	BK	50	*4*	*1*	0	45	98.0 [89.5-99.6]	*80.0 [37.6-96.4]*	100 [92.1-100]	0.88
	VK	50	*4*	*1*	0	45	98.0 [89.5-99.6]	*80.0 [37.6-96.4]*	100 [92.1-100]	0.88
**NG**	BK	50	*4*	*0*	0	46	100 [92.9-100]	*100 [51.0-100]*	100 [92.3-100]	1
	VK	50	*4*	*0*	1	45	98.0 [89.5-99.6]	*100 [51.0-100]*	97.8 [88.7-99.6]	0.88
**MG**	BK	50	8	2	0	40	96.0 [67.6-100]	80 [49.0-94.3]	100 [91.2-100]	0.86
	VK	50	8	2	0	40	96.0 [67.6-100]	80 [49.0-94.3]	100 [91.2-100]	0.86
**TV**	BK	50	*0*	*3*	0	47	94.0 [83.8-97.9]	*0.00 [0.00-56.1]*	100 [92.4-100]	0.00
	VK *	50	*1*	*2*	0	47	96.0 [86.5-98.9]	*33.3 [6.1-79.2]*	100 [92.4-100]	0.48

BK, Bosphore® STD Urethritis Mini Bundle kit; VK, Viasure® Sexually Transmitted Diseases Real Time PCR Detection kit.
CI, confidence interval; CT, *C. trachomatis*; NG, *N. gonorrhoeae*; MG, *M. genitalium*; TV, *T. vaginalis*.
Data in italic are relative to only five or less samples detected positive using the Cobas CT/NG and Cobas TV/MG reference methods.
* The VK has not been approved for use for the detection of *T. vaginalis* in first-void urine.

Table S5. Clinical performance of the two evaluated commercial kits for 25 rectal swabs compared to the reference kits (Cobas CT/NG and Cobas TV/MG)

Pathogens	Evaluated kits	No. of validated samples	Positive with reference		Negative with reference		Overall % agreement [95%CI]	Positive % agreement [95%CI]	Negative % agreement [95%CI]	κ
			Positive	Negative	Positive	Negative				
**CT**	BK	25	9	2	0	14	92.0 [75.0-97.8]	81.8 [52.3-94.9]	100 [78.5-100]	0.83
	VK	25	8	3	0	14	88.0 [70.0-95.8]	72.7 [43.4-90.3]	100 [78.5-100]	0.75
**NG**	BK	25	13	1	0	11	96.0 [80.5-99.3]	92.9 [68.5-98.7]	100 [74.1-100]	0.92
	VK*	25	12	2	0	11	92.0 [75.0-97.8]	85.7 [60.1-96.0]	100 [74.1-100]	0.84
**MG**	BK	25	*3*	*0*	1	21	96.0 [80.5-99.3]	*100 [43.9-100]*	95.5 [78.2-99.2]	0.83
	VK	25	*3*	*0*	0	22	100 [86.7-100]	*100 [43.9-100]*	100 [85.1-100]	1
**TV**	BK	25	*0*	*0*	1	24	96.0 [80.5-99.3]	nc	96.0 [80.5-99.3]	0.00
	VK	25	*0*	*0*	0	25	100 [86.7-100]	nc	100 [86.7-100]	nc

BK, Bosphore® STD Urethritis Mini Bundle kit; VK, Viasure® Sexually Transmitted Diseases Real Time PCR Detection kit.
CI, confidence interval ; CT, *C. trachomatis*; nc, non calculable ; NG, *N. gonorrhoeae* ; MG, *M. genitalium* ; TV, *T. vaginalis*.
Data in italic are relative to only three or less samples detected positive using the Cobas CT/NG and Cobas TV/MG reference methods.
* None of the kits have been approved for use with rectal swab samples, except the VK for the detection of *N. gonorrhoeae* using rectal swabs.

Table S6. Clinical performance of the evaluated commercial two kits for 24 throat swabs compared to the reference kits (Cobas CT/NG and Cobas TV/MG)

**Pathogens**	**Evaluated kits**	**No. of validated samples**	**Positive with reference**		**Negative with reference**		**Overall % agreement [95%CI]**	**Positive % agreement [95%CI]**	**Negative % agreement [95%CI]**	**κ**
			**Positive**	**Negative**	**Positive**	**Negative**				
**CT**	BK	24	*2*	*0*	0	22	100 [86.2-100]	*100 [34.2-100]*	100 [85.1-100]	1
	VK	24	*2*	*0*	0	22	100 [86.2-100]	*100 [34.2-100]*	100 [85.1-100]	1
**NG**	BK	24	9	9	0	6	62.5 [42.7-78.8]	50.0 [29.0-71.0]	100 [61.0-100]	0.33
	VK	24	10	8	0	6	66.7 [46.7-82.0]	55.6 [33.7-75.4]	100 [61.0-100]	0.38
**MG**	BK	24	*1*	*1*	0	22	95.8 [79.8-99.3]	*50 [9.5-90.5]*	100 [85.1-100]	0.65
	VK	24	*2*	*0*	0	22	100 [86.2-100]	*100 [34.2-100]*	100 [85.1-100]	1
**TV**	BK	24	*0*	*0*	8	16	66.7 [46.7-82.0]	nc	66.7 [46.7-82.0]	nc
	VK	24	*0*	*0*	0	24	100 [86.2-100]	nc	100 [86.2-100]	nc

BK, Bosphore® STD Urethritis Mini Bundle kit; VK, Viasure® Sexually Transmitted Diseases Real Time PCR Detection kit.
CI, confidence interval ; CT, *C. trachomatis*; nc, non calculable ; NG, *N. gonorrhoeae* ; MG, *M. genitalium* ; TV, *T. vaginalis*.
Data in italic are relative to only two or less samples detected positive using the cobas CT/NG and cobas TV/MG reference methods.
None of the evaluated commercial kits have been approved for use with throat swabs.
